# Supplementary material for: Narratives and counter-narratives in religious responses to COVID-19: A computational text analysis
Source: PLoS One. 2022 Feb 3;17(2):e0262905. doi: 10.1371/journal.pone.0262905 (PMC8812967; doi:10.1371/journal.pone.0262905)
Supplement: S1 Table — (DOCX) [file pone.0262905.s001.docx]

Table S1. Public Health Guidance for Religious Groups from the Centers for Disease Control and Prevention and the World Health Organization

URLs identified July 3, 2020

| Date | Institution | Title of Document | url |
| --- | --- | --- | --- |
| 4/7/20 | WHO | Practical considerations and recommendations for religious leaders and faith-based communities in the context of COVID-19: Interim Guidance | <https://www.who.int/publications/i/item/practical-considerations-and-recommendations-for-religious-leaders-and-faith-based-communities-in-the-context-of-covid-19> |
| 4/7/20 | WHO | Decision tree for risk assessment tool for Religious Leaders and Faith-based Communities in the context of COVID-19 | <https://www.who.int/publications/m/item/decision-tree-for-risk-assessment-tool-for-religious-leaders-and-faith-based-communities-in-the-context-of-covid-19> |
| 4/9/20 | CDC | FAQs for Administrators and Leaders of Community- and Faith-Based Organizations | <https://www.cdc.gov/coronavirus/2019-ncov/downloads/316368B_FS_COVID19_CommunityFaithBased.pdf> |
| 4/14/20 | WHO | Staying safe during season of religious and cultural events, amid the COVID-19 outbreak | <https://www.who.int/westernpacific/news/feature-stories/detail/staying-safe-during-season-of-religious-and-cultural-events-amid-the-covid-19-outbreak> |
| 4/15/20 | WHO | Safe Ramadan practices in the context of the COVID-19 | <https://apps.who.int/iris/bitstream/handle/10665/331767/WHO-2019-nCoV-Ramadan-2020.1-eng.pdf?sequence=1&isAllowed=y> |
| 5/22/20 | CDC | CDC Releases Recommendations for Communities of Faith | <https://www.cdc.gov/media/releases/2020/s0522-cdc-releases-recommendations-faith.html> |
| 5/23/20 | CDC | Interim Guidance for Communities of Faith | <https://www.cdc.gov/coronavirus/2019-ncov/community/faith-based.html> |
| 6/11/20 | CDC | Checklist for Communities of Faith | <https://www.cdc.gov/coronavirus/2019-ncov/community/organizations/checklist.html> |
