# Supplementary figures and images for: Narratives and counter-narratives in religious responses to COVID-19: A computational text analysis
Source: PLoS One. 2022 Feb 3;17(2):e0262905. doi: 10.1371/journal.pone.0262905 (PMC8812967; doi:10.1371/journal.pone.0262905)

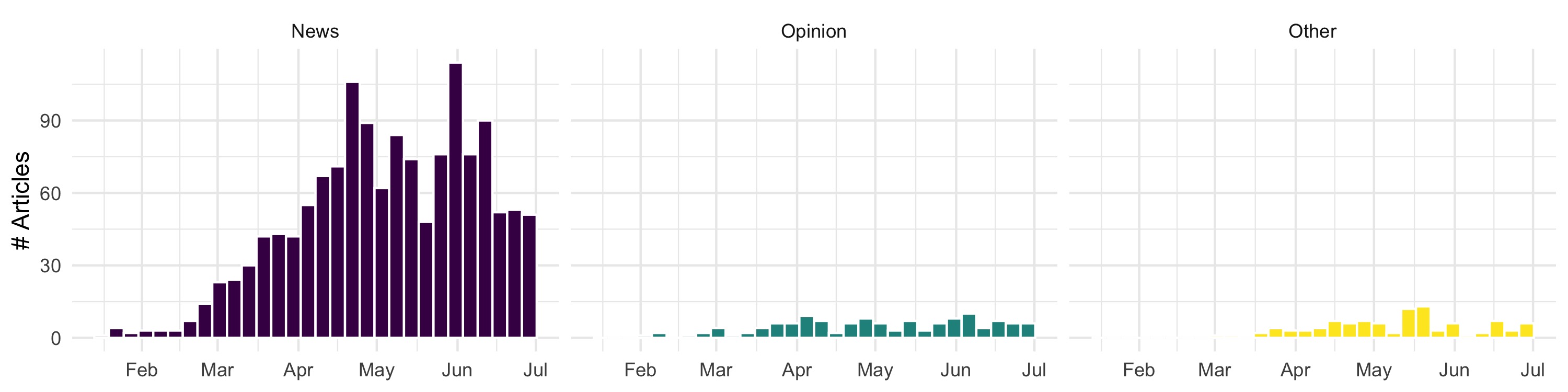

Supplement: S1 Fig — (JPG) [file pone.0262905.s004.jpg]
