# Supplementary material for: Narratives and counter-narratives in religious responses to COVID-19: A computational text analysis
Source: PLoS One. 2022 Feb 3;17(2):e0262905. doi: 10.1371/journal.pone.0262905 (PMC8812967; doi:10.1371/journal.pone.0262905)
Supplement: Attachment — Submitted filename: Response to Reviewers PONE0730.docx [file pone.0262905.s005.docx]

Dear PLoS One Editor and Reviewers,

My co-authors and I thank you for the helpful reviews, and for this opportunity to revise and improve our paper “Narratives and Counter-Narratives in Religious Responses to COVID-19: A Computational Text Analysis”. We have revised the manuscript as suggested. To highlight the most important changes: 1) we have added a new table (Table 1) showing characteristics of the three text corpora; 2) we have added a new table (Table 3) for the regressions that underlie Figure 2; and 3) we have provided a more extensive description of the methods we used. Following are specific responses to the comments we received:

Reviewer 1

1. *The reviewer summarizes our findings to say that we find a gap between religion and science towards the COVID pandemic.*

We think the actual findings are more complicated. Our findings from the analysis of topic models showed that there was not a gap, but actually a very close association between the official statements of religious groups, and the science-based recommendations of the World Health Organization (WHO) and Centers for Disease Control and Prevention (CDC). In the topic model analysis there was hardly any difference in the order of the most important topics for these two bodies of text (religion and science), while there was little similarity at all for the top topics of the New York Times (media) compared with the other two. So it would be most accurate to say that the gap in the topic modeling was between the media representations, and those of both science and religion, which did not differ from each other.

Where we also saw a gap, at least initially, was in the sentiment analysis, where we compared the sentiments of the religious group statements with the sentiments of media reports in the New York Times about religion and COVID. In this analysis the New York Times began with strongly negative sentiment that trended positive over the period. There was certainly a gap in the early part of the period when compared with the always-positive sentiment scores of the faith organizations. But the New York Times trend brought its sentiment score into positive range, and by the end of the period the confidence intervals overlapped with the faith organizations within the positive sentiment range.

*The data sources are heterogeneous.*

To address this, we have added a new Table 1 to summarize the characteristics of the data sets (lines 148-150). The first issue concerning heterogeneity that the reviewer raises is about the writing style of public health guidance compared with the journalism of the New York Times. To address this, we have added some references for other studies that have similarly performed computational text analysis on text from different types of sources (lines 139-146).

Importantly, however, we would like to underscore that all three sources were written for the public to read. They were all meant as health information communications for broad audiences, and it was necessary for all of them to be accessible to a large number of readers. The Flesch-Kincaid Grade Level is a measure commonly used to assess the reading level of text by assessing sentence length and word length (<https://readable.com/blog/the-flesch-reading-ease-and-flesch-kincaid-grade-level/>). The reading level for the New York Times is 10^th^ grade (<https://www.fullmedia.com/how-do-you-measure-readability>). The reading level for CDC documents was studied with respect to H1N1 flu – it showed that their guidance documents meant for “multiple audiences” were at the 8^th^-10^th^ grade levels (<https://www.researchgate.net/publication/51753970_How_Accessible_Was_Information_about_H1N1_Flu_Literacy_Assessments_of_CDC_Guidance_Documents_for_Different_Audiences/figures?lo=1>). Reading levels for WHO guidance on COVID were assessed at a range of 8^th^ grade to 18^th^ grade (<https://onlinelibrary.wiley.com/doi/10.1111/1753-6405.13066>), but this included WHO documents for all audiences, and did not break them down into those meant for lay audiences such as religious groups or the general public. Thus we would argue that the commonality of the documents with respect to their intended general audiences and reading levels reduces their heterogeneity.

*A second issue raised by the reviewer relating to heterogeneity is the difference in the sizes of the three bodies of text.*

Table 1 (new) shows the word count for each of the corpora, which are certainly different for the total word count, but actually quite similar with respect to the average word count per document among the three sources (lines 148-150). However, and more importantly, the two methods we use to analyze the data are independent of the size of the document. Our topic modeling depended on the relative frequency of the appearance of topics in three bodies of text – we compared these in Table 2 (Table 1 in original). Our sentiment analysis takes the sentence as the unit of analysis and compares the average sentiment score across sentences. Thus both analyses are independent of the length or number of the documents in the three bodies of text.

Thank you for prompting us to clarify these issues. We have added text where appropriate to address these concerns (lines 139-146).

2. *The reviewer had no concerns with the statistical analysis.*

3. *The reviewer found that we had provided the sources of all data used.*

4. *Writing issues.*

1) Thank you for pointing out the typo in the p value in (now) Table 2. We have corrected this.

2) The Kendall coefficient is used to test the similarity of ranks. It was thus appropriate to use when assessing the similarity/difference of the rank ordering of the frequency of topics we discovered across the three sources. We have added text to further describe this statistical test for our readers (lines 191-192).

3) We have decided to add a Table 3 (lines 265-267) that shows the regression models that were the underlying basis of Figure 2. (The review says “Figure 3” but the manuscript did not have a Figure 3 so we interpret the reviewer’s comment to refer to Figure 2). We have added this Table 3 to the manuscript; Figure 2 was also revised to include a more detailed Note to facilitate interpretation.

Reviewer 2

1. *Time period of the study.*

Reviewer 2 questions the time period of the study. We agree that the pandemic was ongoing and had not yet reached its second wave during the study period. The relevance of this fact is different for the three text sources.

- Public health. For the WHO guidance, there has been no new guidance issued for faith communities since those we include, so extending the time period would not gain any new documents from this source. The CDC did update its original guidance in February 2021, but much of the material is duplicated, so it is not really new guidance.

- Religion. Faith-based organizations issued their guidance beginning in March 2020. Our corpus extends only through early July. But even by then, some religious bodies were issuing second and third statements that were not amendments of their initial statements, but were more targeted and specific to narrower concerns or member groups. Where there were multiple statements available from one faith-based organization, we always included only the first or most general statement of the group’s guidance for individual member organizations, and importantly, we included only one per group. Thus extending the time period here would likely only yield more of the type of duplicative documents that we have already been excluding.

- Media. The source for which this concern might be most relevant would be for the New York Times, for which there would be ongoing coverage of religion and COVID. However, given the somewhat fixed nature of the other two corpora with the beginning of the pandemic, it would not be appropriate to allow a longer time period for just one source and not the other two.

2. *Consider adding other news sources.*

We have discussed this possibility, and agree that it would be useful to do so, but only in a later paper where we could take a different approach to comparing, for example, media reports on religion and COVID in different regions of the US. Given that the New York Times has national status as “the paper of record” and was simultaneously the local paper for the worst initial outbreak of the epidemic in the US, it seems more than adequate for our comparisons with the other sources. The New York Times is also very user-friendly for the sort of investigation we are conducting.

3. *Provide more details of the methods used for analysis*.

We have provided additional detail for readers perhaps unfamiliar with the new computational text analysis methods of topic modeling (lines 181-185) and sentiment analysis (lines 154-171) in the Methods section of the text.

We greatly appreciate this opportunity to revise and improve our paper for the readers of PLoS One, and we look forward to your response.

Sincerely,

Ellen Idler, John Bernau, Dimitrios Zaras

Emory University
