# Supplementary material for: Narratives and counter-narratives in religious responses to COVID-19: A computational text analysis
Source: PLoS One. 2022 Feb 3;17(2):e0262905. doi: 10.1371/journal.pone.0262905 (PMC8812967; doi:10.1371/journal.pone.0262905)
Supplement: Attachment — Submitted filename: Response to reviewers R2.docx [file pone.0262905.s006.docx]

Response to reviewers R2

Reviewer 1 and Reviewer 2 have answered all 5 questions with “Yes”, indicating approval of the manuscript.

Reviewer 1

Thank you for your kind words. We are glad that our clarification of the heterogeneity of the sources of text, and the length of the documents meets with your approval. We have added an additional, very recent, reference that addresses computational text analysis of documents from different sources (Abdo et al. 2021).

Reviewer 2

Thank you for your approval of our revisions.

We have now added specific examples of sentiment polarity scores that show the modification of adjectives (lines 177-180). The examples are taken from an authoritative source on the analytic method.

We have also added formulae to provide more detail on the Kendall’s coefficient, which we used to test the similarity of the rank order of the topics in the three sources (lines 213-229).

In addition, we would note that two sections of text are highlighted because they were edited for clarity (Abstract lines 27-45, and Results lines 312-322). No substantive changes were made.

Thank you all for your care and consideration.
